# Yield of solid AFB culture in addition to automated liquid culture for diagnosis of mycobacterial infections

**DOI:** 10.1128/jcm.01551-24

**Published:** 2024-11-27

**Authors:** Dongsheng Han, Niaz Banaei

**Affiliations:** 1 Department of Pathology, Stanford University School of Medicine 10624 , Stanford, California, USA; 2 Department of Pathology, The First Affiliated Hospital of Zhejiang University School of Medicine 71069 , Hangzhou, China; 3 Clinical Microbiology Laboratory, Stanford Health Care 474436 , Stanford, California, USA; 4 Division of Infectious Diseases & Geographic Medicine, Stanford University School of Medicine 10624 , Stanford, California, USA; The University of North Carolina at Chapel Hill School of Medicine, Chapel Hill, North Carolina, USA

**Keywords:** *Mycobacterium tuberculosis*, non-tuberculous mycobacteria, AFB culture, liquid culture, solid culture

## LETTER

Acid-fast bacillus (AFB) cultures are crucial for diagnosing and managing tuberculosis (TB) and non-tuberculous mycobacteria (NTM) infections. Expert guidelines recommend both liquid and solid AFB cultures for all specimens from patients with suspected TB and NTM infections (1, 2). Prior studies on the added value of solid culture alongside automated liquid systems have yielded conflicting results. Some suggest standalone liquid culture is sufficient and eliminating solid culture would not diminish the clinical actionability of AFB culture results (3, 4), while others argue for the necessity of both cultures to maximize sensitivity (5, 6). However, the latter studies had limitations as they did not evaluate the clinical actionability of liquid-negative/solid-positive culture results.

We conducted a retrospective analysis on 31,371 mycobacterial cultures submitted to the Stanford Health Care clinical microbiology laboratory between 2020 and 2024. All specimens were concurrently cultured using Middlebrook 7H11/S7H11 agar (solid) and mycobacterial growth indicator tubes broth (liquid) media. We assessed the proportion of liquid-positive/solid-negative cultures taking into account the species recovered, sample type, and clinical actionability of results. Clinical data were extracted from electronic medical records. The study design, definitions, and results are detailed in the supplementary material.

**Fig 1:**
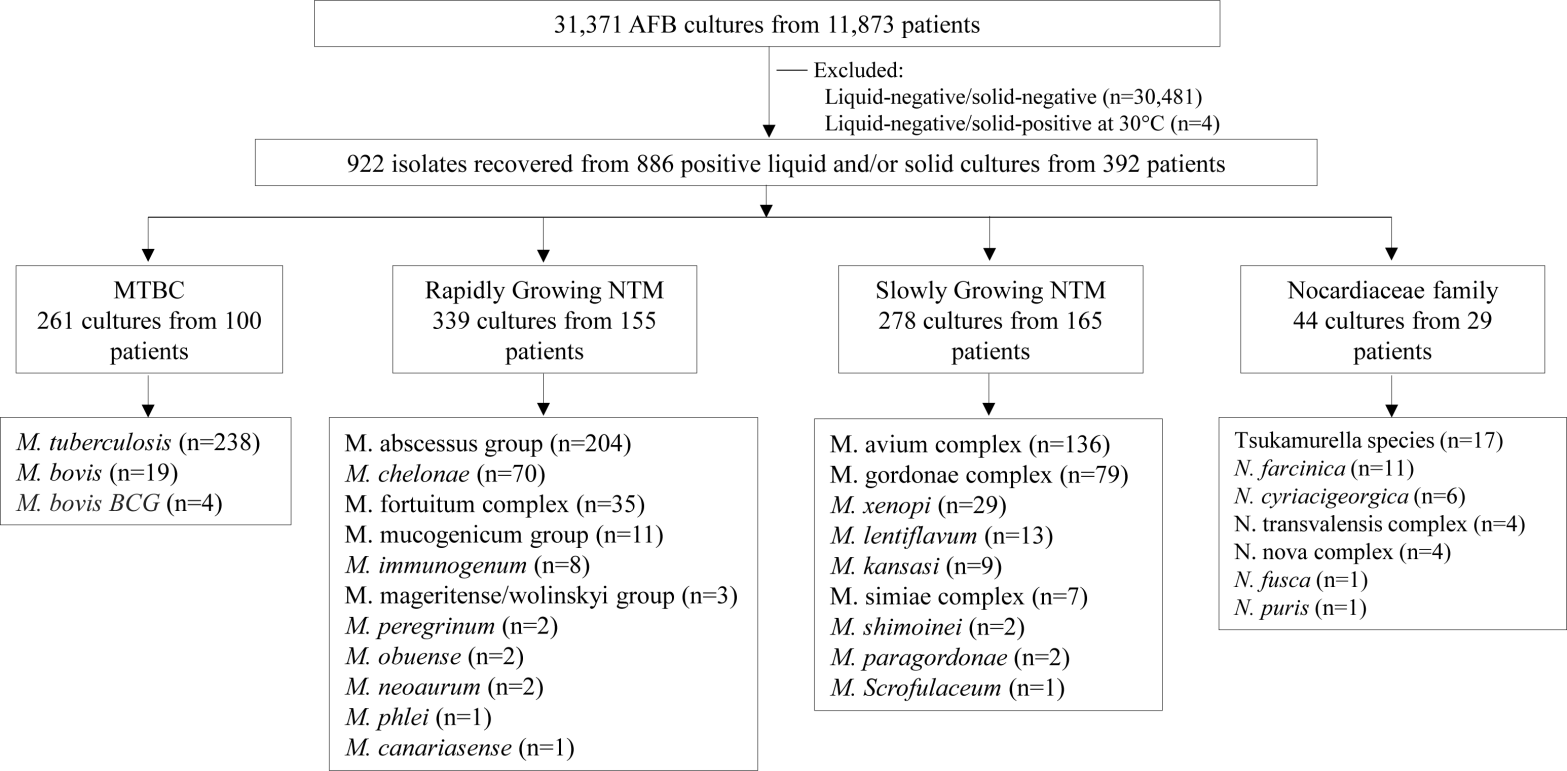
Schematic overview of positive liquid and solid cultures included in this study.

A total of 922 positive AFB cultures were identified, with 433 (47.0%) liquid-positive/solid-positive, 316 (34.3%) liquid-positive/solid-negative, and 173 (18.8%) liquid-negative/solid-positive (Table 1). The organisms isolated included 261 *Mycobacterium tuberculosis* complex (MTBC), 617 NTM consisting of 339 rapid-growing and 278 slow-growing mycobacteria, and 44 *Nocardiaceae* (Fig. 1). In 32 cultures, ≥2 organisms were recovered. Among the 261 MTBC cultures, 140 were clinically actionable, with 6 (4.3%) from five nonsterile sources and one tissue specimen from six patients with new TB diagnoses being liquid-negative/solid-positive (Table 1). Of the 617 NTM cultures, 212 were clinically actionable, including 7 (1.1%) from three sputa and four sterile specimens from seven patients being liquid-negative/solid-positive (Table 1). Notably, among the three sputa, only one (0.2%) growing *M. abscessus* group led to a new NTM diagnosis. The other two (0.3%) came from patients with prior NTM infections, which grew *M. abscessus* and *M. kansasii*, respectively, resulting in treatment changes. Aerobic actinomycetes liquid-negative/solid-positive cultures were rare (*n* = 6) and not actionable.

**TABLE 1:** Distribution of positive liquid and solid cultures and their clinical significance

Category^*a*^	Overall positive [no. of cultures (%)/no. of patients]			MTC [no. of cultures (%)/no. of patients]			NTM [no. of cultures (%)/no. of patients]			Nocardiaceae [no. of cultures (%)/no. of patients]		
	Sterile source	Nonsterile sources	All sources	Sterile source	Nonsterile sources	All sources	Sterile source	Nonsterile sources	All sources	Sterile source	Nonsterile sources	All sources
Overall	133 (100)/82	789 (100)/327	922 (100)/392	76 (100)/44	185 (100)/70	261 (100)/100	52 (100)/34	565 (100)/254	617 (100)/286	5 (10.0)/5	39 (10.0)/25	44 (10.0)/29
Liquid+/Solid+	77 (57.9)/52	356 (45.1)/177	433 (47.0)/221	45 (59.2)/31	101 (54.6)/44	146 (55.9)/68	29 (55.8)/18	238 (42.1)/124	267 (43.3)/142	3 (6.0)/3	17 (43.6)/13	20 (45.5)/15
Liquid+/Solid−	31 (23.3)/25	285 (36.1)/185	316 (34.3)/207	26 (34.2)/20	68 (36.8)/43	94 (36.0)/60	4 (7.7)/4	200 (35.4)/136	204 (33.1)/140	1 (2.0)/1	17 (43.6)/15	18 (40.9)/16
Liquid−/Solid+	25 (18.8)/23	148 (18.8)/103	173 (18.8)/124	5 (6.6)/5	16 (8.6)/15	21 (8.0)/19	19 (36.5)/17	127 (22.5)/83	146 (23.7)/99	1 (2.0)/1	5 (12.8)/5	6 (13.6)/6
Clinically actionable	81 (60.9)/64	287 (36.4)/173	368 (39.9)/226	54 (71.1)/42	86 (46.5)/68	140 (53.6)/100	23 (44.2)/18	189 (33.5)/100	212 (34.4)/118	4 (8.0)/4	12 (30.8)/8	16 (36.4)/11
Liquid+/Solid+	54 (40.6)/46	162 (20.5)/114	216 (23.4)/155	34 (44.7)/29	40 (21.6)/35	74 (28.4)/59	17 (32.7)/14	114 (20.2)/72	131 (21.2)/86	3 (6.0)/3	8 (20.5)/7	11 (25)/9
Liquid+/Solid-	22 (16.5)/19	117 (14.8)/87	139 (15.1)/104	19 (25.0)/16	41 (22.2)/37	60 (23.0)/51	2 (3.8)/2	72 (12.7)/49	74 (12.0)/51	1 (20.0)/1	4 (10.3)/3	5 (11.4)/4
Liquid-/Solid+	5 (3.8)/5	8 (1.0)/8	13 (1.4)/13	1 (1.3)/1	5 (2.7)/5	6 (2.3)/6	4 (7.7)/4	3 (0.5)/3	7 (1.1)/7	0	0	0
New diagnosis	74 (55.6)/61	156 (19.8)/118	230 (24.9)/168	54 (71.1)/42	70 (37.8)/65	124 (47.5)/97	16 (30.8)/15	75 (13.3)/48	91 (14.7)/63	4 (80.0)/4	11 (28.2)/7	15 (34.1)/10
Liquid+/Solid+	49 (36.8)/43	78 (9.9)/67	127 (13.8)/105	34 (44.7)/29	30 (16.2)/59	64 (24.5)/55	12 (23.1)/11	41 (7.3)/30	53 (8.6)/41	3 (60.0)/3	7 (17.9)/6	10 (22.7)/8
Liquid+/Solid−	21 (15.8)/18	72 (9.1)/61	93 (10.1)/77	19 (25.0)/16	35 (18.9)/33	54 (20.7)/47	1 (1.9)/1	33 (5.8)/26	34 (5.5)/27	1 (20.0)/1	4 (10.3)/3	5 (11.4)/4
Liquid−/Solid+	4 (3.0)/4	6 (0.8)/6	10 (1.1)/10	1 (1.3)/1	5 (2.7)/5	6 (2.3)/6	3 (5.8)/3	1 (0.2)/1	4 (0.6)/4	0	0	0
Treatment response	7 (5.3)/7	131 (16.6)/80	138 (15.0)/87	0	16 (8.6)/12	16 (6.1)/12	7 (13.5)/7	114 (20.2)/67	121 (19.6)/74	0	1 (2.6)/1	1 (2.3)/1
Liquid+/Solid+	5 (3.8)/5	84 (10.6)/58	89 (9.7)/63	0	10 (5.4)/9	10 (3.8)/9	5 (9.6)/6	73 (12.9)/48	78 (12.6)/53	0	1 (2.6)/1	1 (2.3)/1
Liquid+/Solid−	1 (0.8)/1	45 (5.7)/37	46 (5.0)/38	0	6 (3.2)/6	6 (2.3)/6	1 (1.9)/5	39 (6.9)/31	40 (6.5)/32	0	0	0
Liquid−/Solid+	1 (0.8)/1	2 (0.3)/2	3 (0.3)/3	0	0	0	1 (1.9)/1	2 (0.4)/2	3 (0.5)/3	0	0	0
Clinically non-actionable	52 (39.1)/37	502 (63.6)/238	554 (60.0)/273	22 (28.9)/14	99 (53.5)/37	121 (46.3)/49	29 (55.8)/34	376 (66.5)/192	405 (65.6)/808	1 (20.0)/1	27 (69.2)/19	28 (63.6)/20
Liquid+/Solid+	23 (17.3)/15	194 (24.6)/102	217 (23.5)/117	11 (14.5)/8	61 (33.0)/30	72 (27.6)/38	12 (23.1)/18	124 (21.9)/67	136 (22.0)/74	0	9 (23.1)/7	9 (20.5)/7
Liquid+/Solid−	9 (6.8)/8	168 (21.3)/128	177 (19.2)/135	7 (9.2)/6	27 (14.6)/16	34 (13.0)/21	2 (3.8)/4	128 (22.7)/103	130 (21.1)/105	0	13 (33.3)/12	13 (29.5)/12
Liquid−/Solid+	20 (15.0)/18	140 (17.7)/96	160 (17.4)/113	4 (5.3)/4	11 (5.9)/10	15 (5.7)/13	15 (28.8)/17	124 (21.9)/81	139 (22.5)/94	1 (20.0)/1	5 (12.8)/5	6 (13.6)/6

^*a*^ +, positive; −, negative.

These results show that solid culture led to diagnosis of TB in some patients but rarely added value to liquid culture for nonsterile specimens in patients with pulmonary NTM infection. Our findings partly concur with conclusions of two prior studies stating that eliminating solid culture would be acceptable given their low yield and low clinical actionability (3, 4). For clinical laboratories in low TB incidence countries where a great majority of AFB cultures are performed on respiratory samples to rule out NTM infection, the elimination of solid AFB culture has implications for labor and reagent cost savings, as well as for more effective use of laboratory resources given the staffing shortages (7).

In summary, liquid-negative/solid-positive cultures made up a small fraction of AFB cultures and were rarely actionable for NTM. Elimination of solid AFB culture for nonsterile respiratory samples from patients with suspected NTM infection has implications for cost savings and laboratory quality.

## SUPPLEMENTAL MATERIAL

Supplemental material jcm.01551-24-s0001.docx Supplemental methods, Tables S1 to S8, and Fig. S1.

ASM does not own the copyrights to Supplemental Material that may be linked to, or accessed through, an article. The authors have granted ASM a non-exclusive, world-wide license to publish the Supplemental Material files. Please contact the corresponding author directly for reuse.
