# Supplementary material for: Yield of solid AFB culture in addition to automated liquid culture for diagnosis of mycobacterial infections
Source: J Clin Microbiol. 2024 Nov 27;63(1):e01551-24. doi: 10.1128/jcm.01551-24 (PMC11784161; doi:10.1128/jcm.01551-24)
Supplement: Supplemental material — Supplemental methods, Tables S1 to S8, and Fig. S1. [file jcm.01551-24-s0001.docx]

**Supplementary Material**

Yield of Solid AFB Culture in Addition to Automated Liquid Culture for Diagnosis of Mycobacterial Infections

Dongsheng Han and Niaz Banaei

**Materials and Methods**

**Study design.** A retrospective study was conducted at Stanford Health Care clinical microbiology laboratory serving adult and pediatric patients. From December 2020 to April 2024, AFB cultures positive for mycobacteria and aerobic actinomycetes were classified as liquid-positive/solid-positive, liquid-positive/solid-negative, and liquid-negative/solid-positive. Positive solid cultures incubated at 30 °C with no liquid culture counterpart were excluded. The primary endpoint of this study was the liquid-negative/solid-positive proportion and the absolute number of AFB cultures from sterile and nonsterile sources growing MTBC, NTM, and aerobic actinomycetes that were clinically actionable. The schematic overview of study design is shown in supplementary Figure 1.

**Clinical and Laboratory Data.** An electronic report was used to identify patients with positive AFB cultures and their corresponding species, and classified each one as liquid-positive/solid-positive, liquid-positive/solid-negative, and liquid-negative/solid-positive. MTBC-positive cultures were included from December 2020 to April 2024, NTM and aerobic actinomycetes-positive cultures except for *Mycobacterium avium* complex (MAC) were included from July 2022 to April 2024, and MAC-positive cultures were included from December 2023 to April 2024. The electronic medical records were reviewed to collect data on demographics, underlying disease, radiographic findings, microbiological results, and antibiotic treatment. Each culture result was considered clinically actionable if it established a new diagnosis or was used to assess treatment response using definitions presented in Supplementary Table 1. In patients with positive NTM cultures from respiratory sources, the American Thoracic Society and Infectious Disease Society of America (ATS/IDSA) NTM diagnostic guidelines were applied to determine if they met definition of definite NTM infection (1). Briefly, microbiological criteria for NTM infection included 2 positive sputum cultures or 1 positive culture from bronchioalveolar lavage (BAL); clinical criteria included manifestations of pulmonary infection such as cough or systematic symptoms; imaging criteria included chest CT findings of nodular or cavitary opacities, or x-ray findings of bronchiectasis with multiple small nodules.

**AFB cultures.** Per expert guidelines, 3 sputa were submitted from patients with suspected pulmonary TB and NTM (2, 3). Nonsterile samples such as sputa and BAL were decontaminated and concentrated per standard laboratory procedure (4). Sterile tissues were homogenized, and sterile body fluids were concentrated. Processed samples were concurrently cultured in mycobacterial growth indicator tubes (MGIT) broth (Becton Dickinson, Franklin Lakes, NJ) and on Middlebrook 7H11/S7H11 agar (Hardy Diagnostics, Santa Maria, CA) per manufacturer instructions and incubated for 6 weeks at 37 °C. Positive mycobacterial cultures were identified with a laboratory-developed multiplex PCR (5), matrix-assisted laser desorption/ionization-time of flight mass spectrometry (MALDI-TOF) (Bruker, Billerica, MA), or bacterial 16S rRNA gene sequencing.

**Statistical Analysis**. The Chi-squared test was used for differences between proportions and the Wilcoxon rank sum test was used to compare medians between groups. All statistical tests were computed for a two-sided type I error rate of 5%.

**References**

1. Daley, C. L., J. M. Iaccarino, C. Lange, E. Cambau, R. J. Wallace, C. Andrejak, E. C. Böttger, J. Brozek, D. E. Griffith, L. Guglielmetti, G. A. Huitt, S. L. Knight, P. Leitman, T. K. Marras, K. N. Olivier, M. Santin, J. E. Stout, E. Tortoli, J. van Ingen, D. Wagner, and K. L. Winthrop. 2020. Treatment of Nontuberculous Mycobacterial Pulmonary Disease: An Official ATS/ERS/ESCMID/IDSA Clinical Practice Guideline. Clinical Infectious Diseases 71:905-913.

2. Clinical and Laboratory Standards Institute. Laboratory Detection and Identification of Mycobacteria. 2nd ed. CLSI guideline M48. Wayne, PA: Clinical and Laboratory Standards Institute; 2018.

3. Lewinsohn, D. M., M. K. Leonard, P. A. LoBue, D. L. Cohn, C. L. Daley, E. Desmond, J. Keane, D. A. Lewinsohn, A. M. Loeffler, G. H. Mazurek, R. J. O Brien, M. Pai, L. Richeldi, M. Salfinger, T. M. Shinnick, T. R. Sterling, D. M. Warshauer, and G. L. Woods. 2017. Official American Thoracic Society/Infectious Diseases Society of America/Centers for Disease Control and Prevention Clinical Practice Guidelines: Diagnosis of Tuberculosis in Adults and Children. Clinical Infectious Diseases **64:**e1-e33.

4. Carroll KC, Pfaller MA. 2019. Manual of clinical Microbiology. 12th edition. ASM Press. https://doi.org/10.1128/9781555819842.

5. Richardson, E. T., D. Samson, and N. Banaei. 2009. Rapid Identification of Mycobacterium tuberculosis and Nontuberculous Mycobacteria by Multiplex, Real-Time PCR. Journal of Clinical Microbiology **47:**1497-1502.

**Supplementary Table 1.** Criteria used to categorize clinical impact of culture results in this study.

| **Impact Category** | **Impact Type** | **Definition** |
| --- | --- | --- |
| Clinically Actionable | New Diagnosis | Positive culture result serves as evidence to establish a new TB or NTM infection diagnosis. Examples: (1) The first and only positive M. tuberculosis culture result in a respiratory sample, (2) the first and only positive mycobacterial culture result in a sterile sample such as tissue or body fluid, (3) the first two positive NTM culture results in non-sterile samples such as sputum, (4) the first positive NTM culture results in BAL, and (5) positive culture result is used as evidence to guide treatment plan in a patient with new diagnosis of TB or NTM infection. |
|  | Treatment Response | Positive culture result in a previously diagnosed patient serves as evidence to guide antibiotic treatment plan. |
| Clinically Non-Actionable | None | A positive culture result is considered either (1) contamination or colonization based on provider's impression, or (2) the detected mycobacterial pathogen was not used to make a new diagnosis, to guide treatment plan, or to assess treatment response. |

**Supplementary Table 2.** Patient demographic and clinical features for all patients.

| **Characteristic** | **No. (%) (N=392)** |
| --- | --- |
| **Age, median (IQR)** | 69 (53-77) |
| **Sex, female** | 198 (50.5) |
| **Comorbidities** |  |
| None | 168 (42.9) |
| CF/Bronchiectasis | 71 (18.1) |
| Solid malignancy | 50 (12.8) |
| Diabetes | 23 (5.9) |
| COPD | 18 (4.6) |
| Solid organ transplant | 17 (4.3) |
| Autoimmunity | 9 (2.3) |
| Hematological malignancy | 9 (2.3) |
| HSCT | 6 (1.5) |
| Kidney disease | 6 (1.5) |
| Cardiovascular disease | 3 (0.8) |
| Asthma | 3 (0.8) |
| Other | 9 (2.3) |
| **History of TB/NTM** |  |
| Yes | 120 (30.6) |
| No | 272 (69.4) |
| **Current TB** |  |
| Pulmonary | 55 (14.0) |
| Extrapulmonary | 30 (7.7) |
| Pulmonary and Extrapulmonary | 15 (3.8) |
| **Current NTM** |  |
| Pulmonary | 106 (27.0) |
| Extrapulmonary | 20(5.1) |
| **Specimen type** |  |
| Nonsterile | 789 (85.6) |
| Sputum | 703 (76.2) |
| BAL | 73 (7.9) |
| Gastric fluid | 8 (0.9) |
| Urine | 5 (0.5) |
| Sterile | 133 (14.4) |
| Tissue | 95 (10.3) |
| Body fluid | 30 (3.3) |
| Pleural fluid | 12 (1.3) |
| Peritoneal fluid | 7 (0.8) |
| Synovial fluid | 4 (0.4) |
| Cerebrospinal fluid | 2 (0.2) |
| Other | 5 (0.5) |
| Abscess | 8 (0.9) |

COPD, chronic obstructive pulmonary disease; HSCT, hematopoietic stem cell transplantation; IQR, interquartile range; CF, cystic fibrosis; BAL, bronchoalveolar lavage

**Supplementary Table 3.** Organisms identified in polymicrobial cultures.

| **Organism Combination** | **Count of cultures** |
| --- | --- |
| *M. avium* complex, *M. fortuitum* complex | 3 |
| *M. chelonae*, *M. gordonae* complex | 3 |
| *M. fortuitum* complex, *N. farcinica* | 2 |
| *M. avium* complex, *N. cyriacigeorgica* | 2 |
| *M. abscessus* group, *M. avium* complex | 2 |
| *M. fortuitum* complex, *M. tuberculosis* complex | 2 |
| *M. gordonae* complex, *M. tuberculosis* complex | 2 |
| *M. chelonae*, *M. xenopi* | 1 |
| *M. avium* complex, *M. gordonae* complex | 1 |
| *M. gordonae* complex, *M. xenopi* | 1 |
| *M. kansasii*, *Tsukamurella* species | 1 |
| *M. lentiflavum*, *M. peregrinum* | 1 |
| *M. avium* complex, *Tsukamurella* species | 1 |
| *M. abscessus* group, *M. simiae* complex | 1 |
| *M. abscessus* group, *M. chelonae* | 1 |
| *M. avium* complex, *M. chelonae* | 1 |
| *M. abscessus* group, *M. gordonae* complex | 1 |
| *M. cosmeticum*/*canariasense*, *M. gordonae* complex | 1 |
| *M. tuberculosis* complex, *Tsukamurella* species | 1 |
| *M. abscessus* group, *M. avium* complex, *M. chelonae*, M. fortuitum complex | 1 |
| *M. fortuitum* complex, *M. simiae* complex | 1 |
| *M. chelonae*, *M. tuberculosis* complex | 1 |
| *M. chelonae*, *M. lentiflavum* | 1 |
| Total | 32 |

**Supplementary Table 4.** Species recovered from liquid and or solid cultures.

| **Category** | | **No. of any positive cultures** | **Liquid+/ Solid+**  **No. (%)** | **Liquid+/ Solid- No. (%)** | **Liquid- / Solid+ No. (%)** | ***p* (Liquid+ / Solid- vs. Liquid-/Solid+)** |
| --- | --- | --- | --- | --- | --- | --- |
| MTBC | M. tuberculosis complex | 261 | 146(55.9) | 94(36) | 21(8) | <0.001 |
|  |  |  |  |  |  |  |
| RG NTM | **All RG NTM** | **339** | **161(47.5)** | **102(30.1)** | **76(22.4)** | **0.023** |
|  | *M. abscessus* group | 204 | 122(59.8) | 60(29.4) | 22(10.8) | <0.001 |
|  | *M. chelonae* | 70 | 22(31.4) | 12(17.1) | 36(51.4) | <0.001 |
|  | *M. fortuitum* complex | 35 | 14(40) | 12(34.3) | 9(25.7) | 0.434 |
|  | *M. mucogenicum* group | 11 | 1(9.1) | 9(81.8) | 1(9.1) | <0.001 |
|  | *M. immunogenum* | 8 | 2(25) | 3(37.5) | 3(37.5) | ND |
|  | *M. neoaurum* | 2 | 0 | 1(100) | 1(100) | ND |
|  | *M. mageritense*/*wolinskyi* group | 3 | 0 | 3(100) | 0 | ND |
|  | *M. phlei* | 1 | 0 | 1(100) | 0 | ND |
|  | *M. peregrinum* | 2 | 0 | 0 | 2(100) | ND |
|  | *M. canariasense* | 1 | 0 | 1(100) | 0 | ND |
|  | *M. obuense* | 2 | 0 | 0 | 2(100) | ND |
|  |  |  |  |  |  |  |
| SG NTM | **All SG NTM** | **278** | **106(37.6)** | **102(36.2)** | **70(25.2)** | **0.003** |
|  | *M. avium* complex | 136 | 83(61) | 46(33.8) | 7(5.1) | <0.001 |
|  | *M. gordonae* complex | 79 | 4(5.1) | 21(26.6) | 54(68.4) | <0.001 |
|  | *M. xenopi* | 29 | 10(34.5) | 19(65.5) | 0 | <0.001 |
|  | *M. lentiflavum* | 13 | 1(7.7) | 11(84.6) | 1(7.7) | <0.001 |
|  | *M. kansasi* | 9 | 2(22.2) | 2(22.2) | 5(55.6) | ND |
|  | *M. simiae* complex | 7 | 4(57.1) | 2(28.6) | 1(14.3) | ND |
|  | *M. shimoinei* | 2 | 0 | 0 | 2(100) | ND |
|  | *M. paragordonae* | 2 | 2(100) | 0 | 0 | ND |
|  | *M. scrofulaceum* | 1 | 0 | 1(100) | 0 | ND |
|  |  |  |  |  |  |  |
| Nocardiaceae | **All Nocardiaceae** | **44** | **20(45.5)** | **18(40.9)** | **6(13.6)** | **0.004** |
|  | *Tsukamurella* species | 17 | 4(23.5) | 8(47.1) | 5(29.4) | 0.289 |
|  | *N. farcinica* | 11 | 7(63.6) | 3(27.3) | 1(9.1) | 0.269 |
|  | *N. transvalensis* complex | 4 | 2(50) | 2(50) | 0 | ND |
|  | *N. cyriacigeorgica* | 6 | 4(66.7) | 2(33.3) | 0 | ND |
|  | *N. nova* complex | 4 | 2(50) | 2(50) | 0 | ND |
|  | *N. puris* | 1 | 1(100) | 0 | 0 | ND |
|  | *N. fusca* | 1 | 0 | 1(100) | 0 | ND |
| Total |  | 922 | 433(47.0) | 316(34.3) | 173(18.8) | <0.001 |

+, positive; -, negative; No., number

**Supplementary Table 5.** Positive culture results by sample type.

Other includes 8 gastric fluid and 5 urine; +, positive; -, negative; No., number

**Supplementary Table 6.** Distribution of rapid growing and slow growing NTM positive liquid and solid cultures and their clinical significance.

RG, rapid growing; SG, slow growing; NTM, nontuberculous mycobacteria; +, positive; -, negative; No., number

**Supplementary Table 7.** Distribution of rapid growing and slow growing NTM species growing in liquid-negative/solid-positive cultures and their clinical significance.

| **Category** | | **No. of Liquid-/Solid+ cultures** | **Clinically actionable** | | **Clinically non-actionable, No. of cultures** |
| --- | --- | --- | --- | --- | --- |
|  |  |  | **New**  **Diagnosis,**  **No. of cultures** | **Treatment response, No. of cultures** |  |
| MTBC | M. tuberculosis complex | 21 | 6 | 0 | 15 |
|  |  |  |  |  |  |
| RG NTM | **All RG NTM** | **76** | **3** | **2** | **71** |
|  | *M. abscessus* group | 22 | 1 | 1 | 20 |
|  | *M. chelonae* | 36 | 2 | 0 | 34 |
|  | *M. fortuitum* complex | 9 | 0 | 1 | 8 |
|  | *M. mucogenicum* group | 1 | 0 | 0 | 1 |
|  | *M. immunogenum* | 3 | 0 | 0 | 3 |
|  | *M. neoaurum* | 1 | 0 | 0 | 1 |
|  | *M. mageritense*/*wolinskyi* group | 0 | 0 | 0 | 0 |
|  | *M. phlei* | 0 | 0 | 0 | 0 |
|  | *M. peregrinum* | 2 | 0 | 0 | 2 |
|  | *M. canariasense* | 0 | 0 | 0 | 0 |
|  | *M. obuense* | 2 | 0 | 0 | 2 |
|  |  |  |  |  |  |
| SG NTM | **All SG NTM** | **70** | **1** | **1** | **68** |
|  | *M. avium* complex | 7 | 0 | 0 | 7 |
|  | *M. gordonae* complex | 54 | 0 | 0 | 54 |
|  | *M. xenopi* | 0 | 0 | 0 | 0 |
|  | *M. lentiflavum* | 1 | 0 | 0 | 1 |
|  | *M. kansasi* | 5 | 0 | 1 | 4 |
|  | *M. simiae* complex | 1 | 0 | 0 | 1 |
|  | *M. shimoinei* | 2 | 1 | 0 | 1 |
|  | *M. paragordonae* | 0 | 0 | 0 | 0 |
|  | *M. scrofulaceum* | 0 | 0 | 0 | 0 |
|  |  |  |  |  |  |
| Nocardiaceae | **All Nocardiaceae** | **6** | **0** | **0** | **6** |
|  | *Tsukamurella* species | 5 | 0 | 0 | 5 |
|  | *N. farcinica* | 1 | 0 | 0 | 1 |
|  | *N. transvalensis* complex | 0 | 0 | 0 | 0 |
|  | *N. cyriacigeorgica* | 0 | 0 | 0 | 0 |
|  | *N. nova* complex | 0 | 0 | 0 | 0 |
|  | *N. puris* | 0 | 0 | 0 | 0 |
|  | *N. fusca* | 0 | 0 | 0 | 0 |
| Total |  | 173 | 10 | 3 | 160 |

MTBC, *Mycobacterium tuberculosis* complex; RG, rapid growing; SG, slow growing; NTM, nontuberculous mycobacteria; +, positive; -, negative; No., number

**Supplementary Table 8.** Clinical and laboratory findings of patients with liquid-negative/solid-positive AFB cultures that were clinically actionable.

| **Patient ID** | **Patient history** | **Chest CT Imaging** | **Specimen cultured** | **Culture** | **MTB PCR** | **CFU on solid agar** | **≥3 specimens cultured (No.)** | **ATS NTM classification** | **Clinical impact** |
| --- | --- | --- | --- | --- | --- | --- | --- | --- | --- |
| P38 | 30 female from India with chest pain | Peribronchial and centrilobular nodularity | Sputum, induced | *M. tuberculosis* | Negative | 1 | Yes (3) | NA | Enabled TB diagnosis |
| P43 | 78 female with neuroendocrine carcinoma with weight loss and night sweats | Tree-in-bud pattern and nodular opacities | Sputum, expectorated | *M. tuberculosis* | Negative | 10 | Yes (6) | NA | Enabled TB diagnosis |
| P44 | 76 male from Philippines with chronic cough | Nodules and cavitary lesion | Sputum, induced | *M. tuberculosis* | Not done | 1 | Yes (3) | NA | Enabled TB diagnosis |
| P97 | 1 male from Mexico with cough after TB exposure | Bilateral multifocal consolidation | Gastric fluid | *M. tuberculosis* | Negative | 1 | Yes (4) | NA | Enabled TB diagnosis |
| P30 | 89 male from Myanmar with weight loss with metastatic prostate cancer | Clustered solid nodules | BAL | *M. tuberculosis* | Negative | 3 | No (1) | NA | Enabled TB diagnosis |
| P90 | 68 male from Vietnam with malignant neoplasm of lung | Centrilobular consolidation | Lung biopsy | *M. tuberculosis* | Positive | 1 | No (2) | NA | Enabled TB diagnosis |
| P67 | 72 female with breast cancer and multiple recurrences, and pulmonary MAC and MABS infection | Increased pleural thickening | Sputum, induced | *M. abscessus* group | Not done | 1 | Yes (3) | NTM infection | Therapy restarted |
| P84 | 58 male with AML and skin nodules on lower extremity | ND | Skin biopsy | *M. chelonae* | Not done | 4 | No (1) | NA | Enabled NTM diagnosis |
| P05 | 58 female with bronchiectasis and productive cough | Tree-in-bud pattern and nodules | Sputum, expectorated | *M. abscessus* group | Not done | 10-50 | No (2) | NTM infection | Enabled NTM diagnosis |
| P31 | 75 female with melanoma and surgical site infection | ND | Skin biopsy | *M. chelonae* | Not done | 4 | No (1) | NA | Enabled NTM diagnosis |
| P47 | 29 female with *M. fortuitum* surgical site infection following breast augmentation and mastopexy | ND | Abscess | *M. fortuitum* complex | Not done | 1 | Yes (3) | NA | Escalation of therapy from two drugs to three drugs |
| P25 | 68 male with *M. kansasii* and MAC infection | Enlarging solid nodules bilaterally | Sputum, induced | *M. kansasii* | Negative | 8 | Yes (3) | NTM infection | Therapy restarted |
| P55 | 65 male with ongoing cough and congestion | Scattered nodular opacities | Lung biopsy | *M. shimoinei* | Negative | >100 | Yes (3) | NA | Enabled NTM diagnosis |

MAC, *M. avium* complex; MABS, *M. abscessus* group; AML, acute myeloid leukemia; NA, not applicable; ND, not done; BAL, bronchoalveolar lavage; No., number; NTM, nontuberculous mycobacteria; ATS, American Thoracic Society, MTBC, *M. tuberculosis* complex; CT, computed tomography scan; CFU, colony forming unit; TB, tuberculosis





**Supplementary Figure 1.** Schematic overview of the study design.
